# Atomistic Blueprinting of Electrochemical CO Reduction Reaction Pathways over Transition Metal Phosphides

**DOI:** 10.3390/molecules31081334

**Published:** 2026-04-18

**Authors:** Muhammad Awais, Younes Abghoui

**Affiliations:** Science Institute of the University of Iceland, 102 Reykjavik, Iceland

**Keywords:** chemical physics, DFT, carbon reduction, renewable energy resources, formaldehyde, methanol, methane

## Abstract

Ecosystem disruption is a significant challenge of the contemporary age, arising from substantial CO_2_/CO emissions resulting from dependence on fossil fuels as a primary energy source. Scholars across several fields are striving to mitigate these severe greenhouse gas emissions. The most promising method is to adsorb carbon and convert it into sustainable energy. We sought to diminish CO levels by electrocatalytic reduction using innovative catalytic surfaces, namely transition metal phosphides (TMPs). During this work, VP is recognized as a very effective surface for CO reduction and the synthesis of formaldehyde, methanol, and methane at −0.68 V. Further, hydrogen evolution reaction (HER) does not pose a challenge for any surface, despite all TMPs facilitating CO reduction. In summary, predictions derived from this density functional theory (DFT)-guided analysis provide experimentalists with insights to validate experiments and synthesize active catalysts for CO conversion and green energy generation.

## 1. Introduction

Following the Industrial Revolution, the first signs of an increase in carbon dioxide (CO_2_) levels were discovered. This rise in CO_2_ levels led to a significant deterioration of the climate, which was mostly caused by the overuse of fossil fuels. As a result, the transformation of carbon dioxide into a green fuel by electrochemical conversion is now a major topic of discussion among scientists and has garnered a great deal of interest [1,2,3,4,5]. Carbon dioxide reduction reaction (CO_2_RR) is an efficient technique for the carbon-free synthesis of alcohol and hydrocarbon fuels. This method reduces CO_2_ emissions and fosters the sustainable production of renewable fuels efficiently. CO_2_RR may be conducted by four unique approaches: biochemical, photochemical, electrochemical, and thermochemical. Nevertheless, due to its configurable selectivity and reliable catalytic efficiency, electrochemical CO_2_ reduction ranks among the most effective methods, since this process is exclusively reliant on electrode performance [6,7,8,9,10,11,12,13,14].

Historically, electrochemical CO_2_RRs have used specific metal-based catalysts, with copper being one of the most studied examples. Although copper is an exceptional catalyst for converting CO_2_ into multiple products, it suffers from poor product selectivity. In a recent study, copper was shown to catalyze more than ten products at potentials exceeding −1.0 V, signifying its lack of selectivity and the challenge of product separation [15]. Similarly, another investigation identified copper as an electrode material characterized by both inadequate selectivity and a high overpotential requirement for CO_2_RR [16]. Ultimately, improving activity and selectivity and the tailored design of catalysts are fundamental concerns of the present day. Consequently, researchers are focused on engineering new surfaces and more reliable reaction pathways to develop superior catalysts.

During CO_2_RR, carbon monoxide and formic acid are the principal products that are observed during the initial phase of reduction, resulting from the first proton–electron transfer. However, further proton–electron transition can lead to the formation of hydrocarbons and oxygenates, and this progression often results in a decline of selectivity and proficiency of CO_2_RR [17,18,19,20,21,22,23,24,25]. To overcome this challenge, an improved strategy involves utilizing carbon monoxide (first intermediate of CO_2_RR) for direct electrocatalytic CO reduction (CORR) [26,27]. For instance, MoS_2_, with the doping of transition metal atoms, has been investigated for CORR, and chromium-doped MoS_2_ with vacancies was reported as a promising surface for reducing CO at −0.33 V [28]. Similarly, metal-nitride phosphorene (MN_3_@P) as a single-atom catalyst (SAC) has also been explored for CO reduction [29].

In this research, we have done a comprehensive examination of TMPs as a catalytic surface for the electrocatalytic reduction of CO under room conditions. The structures of TMPs under the current investigation are set in a rocksalt crystalline orientation in (100) facets. The theoretical framework based on DFT is utilized here to assess their catalytic activity. Moreover, these surfaces can show excellent catalytic activities due to their adjustable structures, surprising conductivity and stability under the electrochemical environment [30,31]. It has already been reported that a decrease in thermal dissolution and improvement in the stability of transition metal-based catalysts could be achieved through the introduction of phosphorus atoms [32]. Owing to their superior stability, effective activity, and economic pricing, these materials provide an appealing option for consideration as catalysts [33]. That is the main motive behind the selection of these surfaces in our analysis, and herein their catalytic activity is indicated by free energy diagrams based on their Gibbs free energy values.

## 2. Results and Discussion

### 2.1. Surface Analysis

Following the acquisition of the optimal relaxed structures, which constituted the first phase of our screening process, surface analysis became the subsequent step of the inquiry. Our study primarily focuses on the electrocatalytic reduction of carbon monoxide; hence, we examined the potential of TMPs for CO adsorption and activation. To do this, we adsorbed the CO molecule at two distinct places on each surface, as two potential active sites, such as the metal site (M_TOP_) and the phosphide site (P_TOP_), are investigated to identify an active site for catalytic activity. It is important to mention that, during CO adsorption, we considered two possible configurations of the CO molecule: adsorption through the carbon end and adsorption through the oxygen end. However, on each surface, CO adsorption via the oxygen end was not thermodynamically stable as CO desorbed during structural relaxation. Therefore, in this research, CO adsorption and activation were considered only through CO adsorption via the carbon end. Further information about the active sites is provided by comparing the adsorption energies at the different possible sites in Table 1.

Table 1 indicates that M_TOP_ serves as an active site for all TMPs due to its exergonic CO adsorption energy values in contrast to P_TOP_, hence classifying the metal site as active. Conversely, CrP becomes unstable after CO adsorption, but NbP has an endergonic reaction to CO adsorption. In summary, we excluded NbP and CrP from further research and retained the other five TMPs for further examination in the following sections.

**Figure 1:**
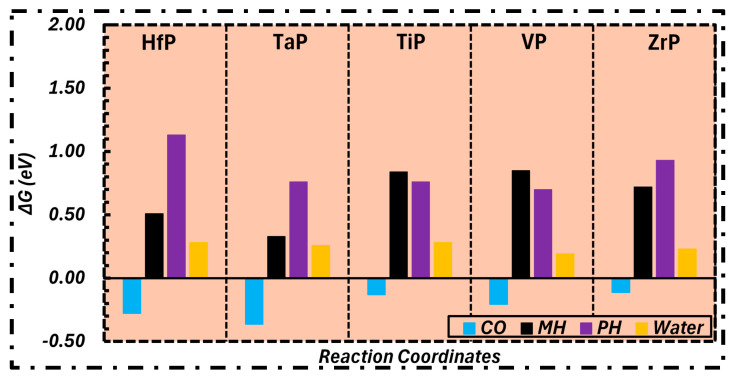
Gibbs free energy values of CO, proton (PH/MH) and water adsorption on TMPs.

After establishing that the active site could adsorb carbon monoxide, we investigated the surface stability and availability of the active site in relation to water contamination under an electrochemical environment. To do this, at the most active site, such as M_TOP_, a water molecule was introduced in order to determine whether or not the active site is dependable for CO adsorption or water adsorption. This was accomplished by analyzing the free energy values, as seen in Figure 1.

The results shown in Figure 1 clearly indicate that adsorption of water is consistently more endergonic than that of CO across all surfaces. Consequently, it is expected that carbon monoxide molecules would occupy the catalyst’s surface before water, hence reducing the likelihood of water-induced surface poisoning. It may be inferred that all catalysts remain readily available and effective for the CORR process.

After this confirmation, TMPs were evaluated for selectivity between CORR and HER, using proton adsorption as a descriptor for hydrogen evolution and comparing it with CO adsorption. The significance of proton adsorption free energy as a descriptor for HER has been previously validated by Nørskov et al. [34] Consequently, we analyze the proton adsorption energy at metal (MH) and phosphide (PH) sites in relation to CO adsorption, as shown in Figure 1. It is evident that the MH and PH values are consistently endergonic compared to those of CO adsorption, indicating that TMPs are preferentially accessible and selective for CORR over HER.

### 2.2. Catalytic Activity Toward CO Reduction

CO reduction may result in the formation of formaldehyde, methanol, and methane after sequential proton–electron transfer steps. It is crucial to emphasize that several potential reaction routes may result in the creation of these compounds, and our study examined all of those possibilities. However, based on the lower energy requirement, we only included the most promising reaction route(s) for the respective product, as included in the free energy landscapes. The most remarkable point is that, in the discussion of the free energy landscapes of each TMP, the best candidate is predicted based on the lowest energy requirement for the reaction. As multiple surfaces are under investigation, the surface that requires the least energy for the formation of formaldehyde, methanol, or methane is considered the most promising one. In other words, the lowest (or smallest) PDS value is considered as a descriptor of higher catalytic efficiency.

**Figure 2:**
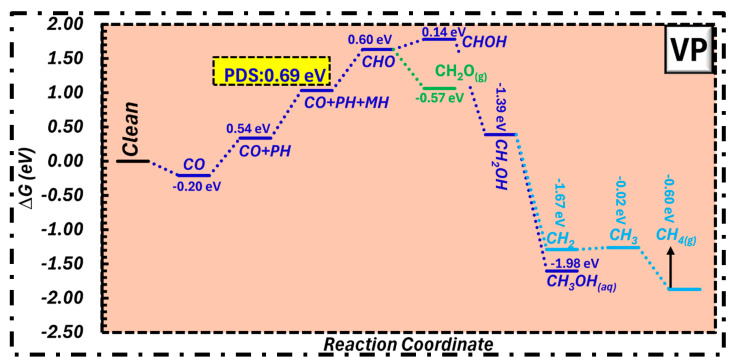
Free energy profile for CORR on the VP surface, exhibiting the catalytic activity for formaldehyde, methanol, and methane production.

Our analysis demonstrated that VP has excellent activity, since it generates formaldehyde at 0.69 eV, as seen in Figure 2. Additionally, the production of both methanol and methane was detected at the adjustable energy of 0.69 eV. Secondly, the PDS during this reaction pathway was observed after the second protonation from CO + PH to CO + PH + MH.

**Figure 3:**
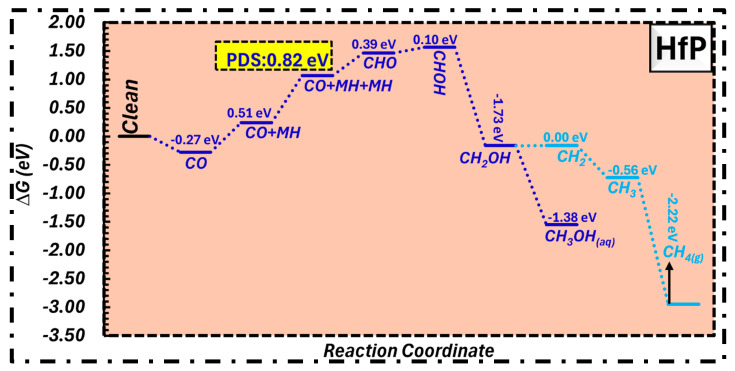
Free energy profile for CORR on the HfP surface, exhibiting the catalytic activity for methanol and methane production.

HfP was also identified as a suitable substrate for the electrocatalytic synthesis of both methanol and methane. As seen in Figure 3, the whole reaction pathway progresses seamlessly, exhibiting the minimal energy requirements of around 0.82 eV for CH_3_OH_(aq)_ and CH_4(g)_. This low energy clearly identifies HfP’s catalytic effectiveness in facilitating the multi-proton–electron CO reduction. The PDS arises at the second proton–electron transfer, especially during the transition from CO + MH to CO + MH + MH.

**Figure 4:**
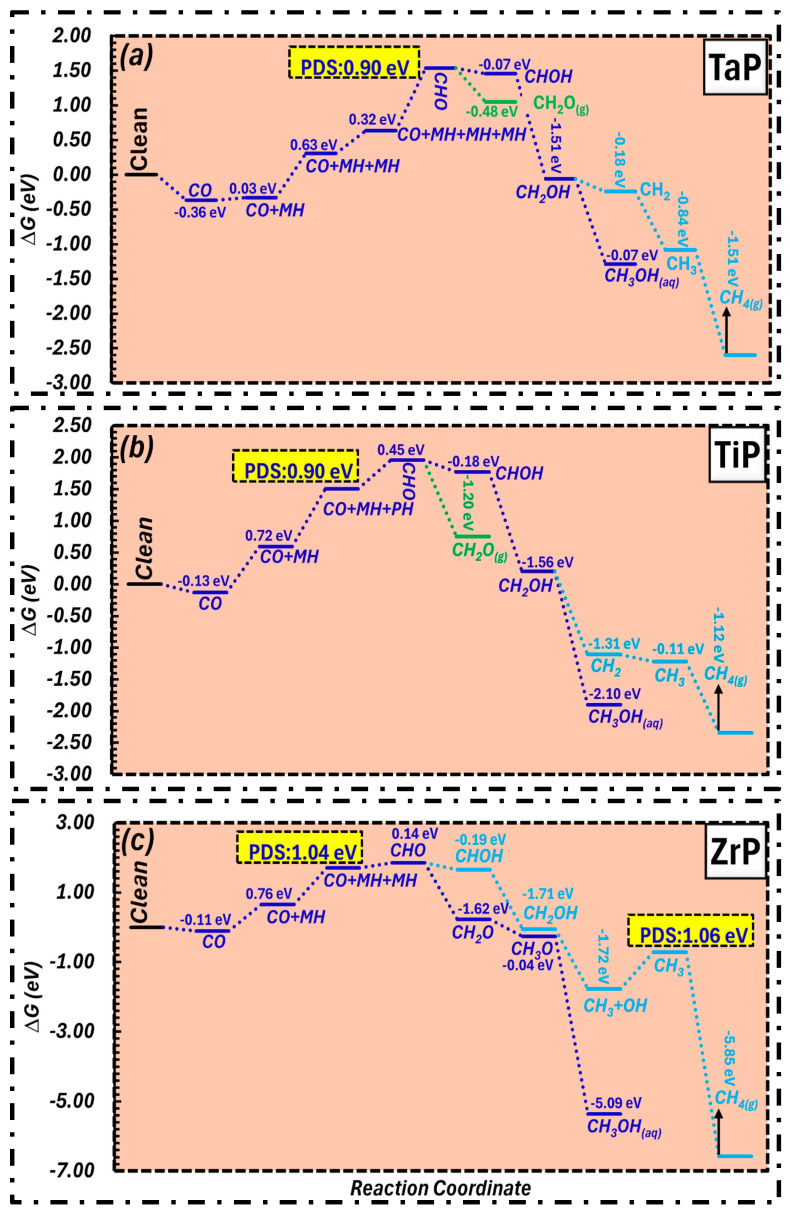
CO reduction pathways for formaldehyde, methanol, and methane formation over (**a**) TaP, (**b**) TiP and (**c**) ZrP.

The surfaces of TaP, TiP and ZrP exhibit good catalytic activity for CO reduction, as shown in Figure 4a–c. This activity enables the generation of environmentally friendly products such as CH_2_O_(g)_, CH_3_OH_(aq)_, and CH_4(g)_ at an energy of 0.90 eV. During the fourth protonation, notably the transition from CHO to CHOH, we were able to see that TaP has the PDS (Figure 4a). However, the PDS for TiP, on the other hand, was seen early after the second protonation step (CO + MH to CO + MH + PH), exhibiting a distinct reaction route while exhibiting equivalent thermodynamic efficiency (Figure 4b). In the meantime, ZrP (Figure 4c) is also capable of reducing CO into distinctive products at a slightly greater energy of 1.04 eV. Furthermore, in a manner comparable to that of TiP, ZrP demonstrates the PDS after the second protonation during the formation of CO+ MH + PH from CO + MH.

**Figure 5:**
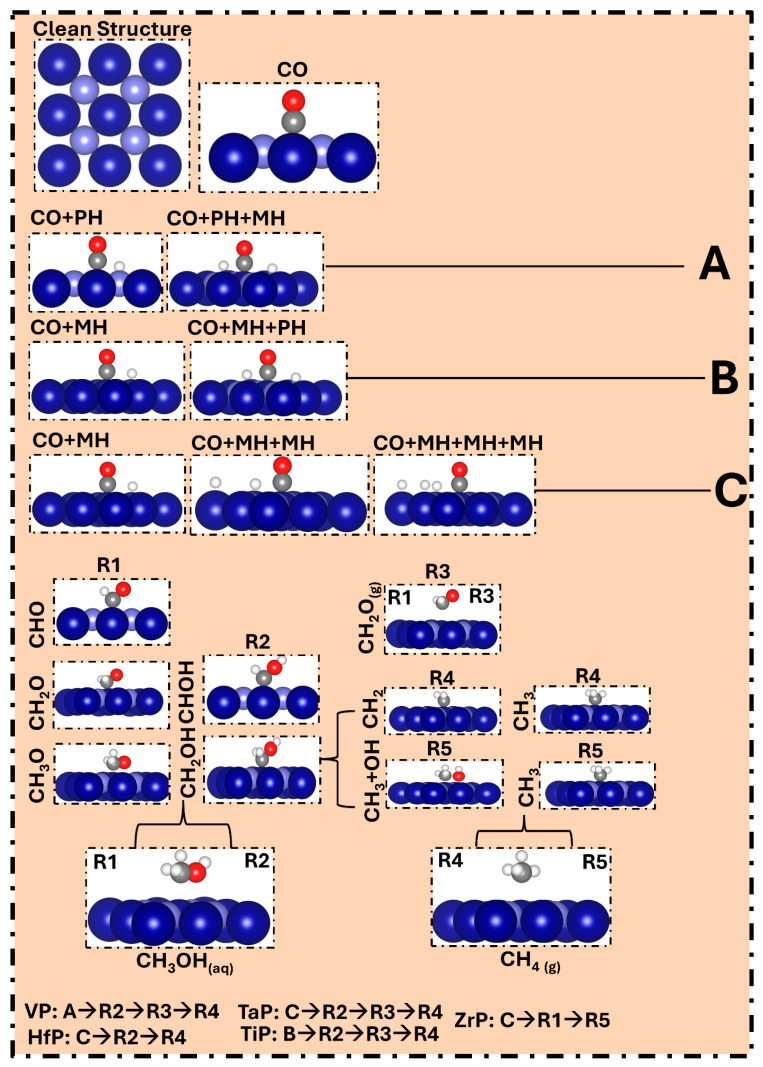
Representation of the reaction intermediates involved in electrochemical CO reduction. Each structure represents the optimized geometry of the corresponding ground state or transition state along the reaction pathway. The reaction pathways A, B and C are classified as general trend observed on different TMPs. At the bottom, the TMPs have been linked to the specific reactions identified during the free-energy profiling. Here, the dark royal-blue spheres represent metal atoms, while the lavender-blue spheres depict the phosphorus atoms. The dark gray spheres represent carbon atoms, the red spheres indicate oxygen atoms and white stands for hydrogen atoms.

To provide a clear and detailed mechanism related to the reaction, including the intermediates, their corresponding orientations and bonding with the catalyst, we prepared Figure 5. In this figure, each FED from Figure 2, Figure 3 and Figure 4 has been illustrated with the assistance of corresponding geometry orientations.

### 2.3. Onset Potentials

**Figure 6:**
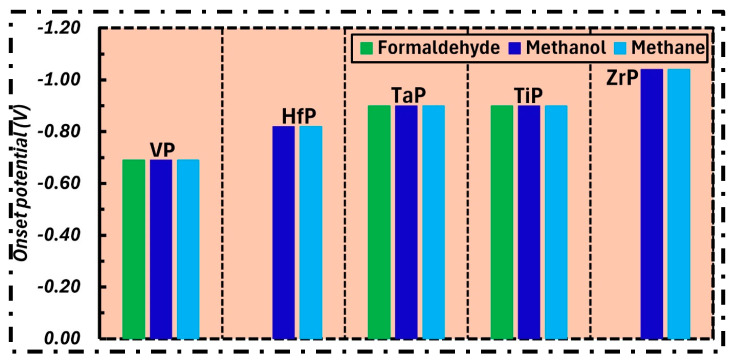
Comparison of onset potentials for formaldehyde, methanol, and methane formation over selected TMP surfaces.

To provide a concise review of our findings, we included a comparative summary of the onset potentials for CO reduction in Figure 6. Although the free energy profiles have already been discussed, this figure provides a clearer overview of the catalytic performance of all five TMPs. As stated above, the PDS was considered a descriptor of catalytic activity. To further improve clarity, the onset potential is also used in this section to evaluate the catalytic activity and identify the best surface. As shown in Figure 6, VP requires a lower (smaller) onset potential for CORR when compared with other TMP surfaces. Therefore, VP is identified as the most promising surface among them.

## 3. Computational Research Details

**Figure 7:**
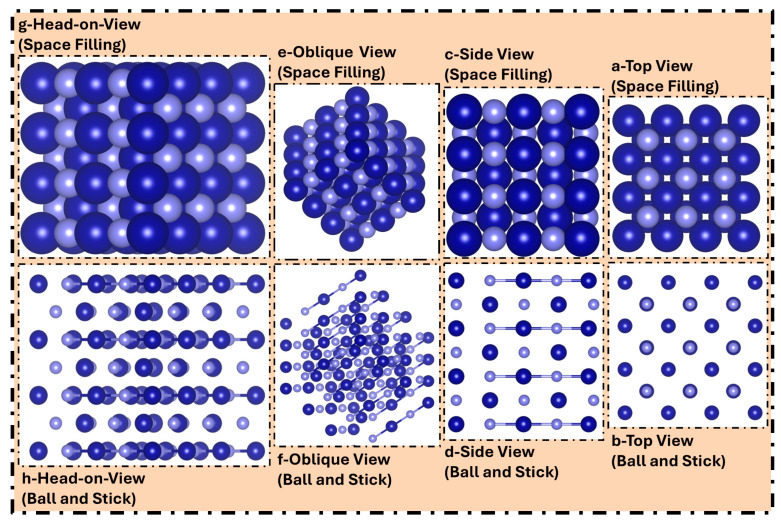
Illustration of various structural arrangements from distinctive viewing directions using space-filling and ball-and-stick models, as employed in this work. (**a**,**b**) Top views, (**c**,**d**) side views, (**e**,**f**) oblique views, (**g**,**h**) head-on views. Herein, dark royal-blue-colored spheres represent the metal atoms, while lavender-blue spheres depict the phosphorus atoms.

In our present investigation, the Vienna Ab initio Simulation Package (VASP 6.4.2) software has been used to examine TMPs as catalytic surfaces for CO reduction under ambient circumstances [35]. The electronic structural analysis was conducted using DFT, with the Revised Perdew–Burke–Ernzerhof (RPBE) as the exchange-correlation functional [36,37]. The computational precision and capabilities of RPBE for the development of certain properties, such as surface analysis and catalytic activity, which are crucial to the present inquiry, prompted its selection. Furthermore, the concordance between experimental findings and computational analyses conducted using RPBE was a primary reason for its selection in our current work [38]. For the optimization, a cut-off energy of 400 eV and a 4 × 4 × 1 Monkhorst-Pack *k*-point grid were used [39]. Secondly, we modified the essential conditions to achieve a relaxed structure with different adsorbates till their atomic forces went below 0.03 eV/Å. Herein, TMP-based electrodes in their (100) crystalline orientation for catalyst design have 40 atoms and five layers, with a 1:1 ratio between the atoms of metal and phosphide, meaning that there are 20 atoms of metal and 20 phosphide atoms. During the investigation, the lower dual layers were restricted, but the remaining three layers were permitted to fully engage with reactants and participate entirely in chemical reactions. To illustrate this, we constructed Figure 7, whereby the borders of the TMPs in the x- and y-axes are treated as periodic, but a vacuum of 20 Å is implemented along the *z*-axis to mitigate the self-interaction of adjacent unit cells.

The reaction pathways identified during the CO reduction into distinctive renewable products on TMPs were investigated using the thermochemical model (TCM), and the effect of applied potential was analyzed using the computational hydrogen electrode (CHE) [40,41]. To compute the adsorption energy ($\Delta E_{ads}$), we used the following equation [6]:

(1) $$\Delta E_{ads}=E_{\left(Total\;system\right)}-E_{\left(System\;without\;adsorbate\right)}-E_{\left(Adsorbed\;species\right)}$$

To calculate the Gibbs free energy, we can use Equation (2)

(2) $$\Delta G=\Delta E_{DFT}+\Delta E_{ZPE}-T\times \Delta S+\Delta G_{pH}+\Delta G_{U}$$

Here

$\Delta E_{DFT}$ = Energy difference between two intermediate states.

$\Delta E_{ZPE}$ = Zero-point energy correction.

ΔS = Change in entropy.

Notably, $\Delta G_{pH}$ indicates the pH shift; the entire value of this component is $2.3\;K_{B}TpH$. K_B_ indicates the Boltzmann constant, T is temperature, and pH represents electrolytic pH; this is independent to overpotential; hence pH is 0 here. That is why $\Delta G_{pH}$ = zero. From Equation (2), we have

$$\Delta G=\Delta E_{DFT}+\Delta E_{ZPE}-T\times \Delta S+(0)+\Delta G_{U}$$

(3) $$\Delta G=\Delta E_{DFT}+\Delta E_{ZPE}-T\times \Delta S+\Delta G_{U}$$

$\Delta G_{U}=-neU$, wherein n represents the number of electrons, e signifies the charge of an electron, and U symbolizes the applied potential. In the absence of applied potential, this term would be equal to 0, and Equation (3) might be represented as:

(4) $$\Delta G=\Delta E_{DFT}+\Delta E_{ZPE}-T\times \Delta S$$

To get information about the energy needed for the reaction, the potential-determining step (PDS) has been determined during the exploration of free energy diagrams. The maximum positive free energy change, as determined by thermodynamic analysis between two consecutive reaction steps, is referred to as the PDS. This reaction step delineates the energy necessary to execute the reaction. PDS values are used to compute the onset potential (OP) for particular products by using the following Equation (5).

(5) $$OP=-\Delta G_{max}/e$$

where $\Delta G_{max}$ reflects the greatest variation in free energy seen between two steps that are next to each other in the reaction route.

## 4. Conclusions

This work used DFT to examine several surfaces composed of transition metals, such as TMPs, for the reduction of CO by electrocatalysis. During the surface investigation, we observed that no surface was in the danger zone of water piousness, since each slab exhibited higher CO adsorption compared to water molecules. Secondly, HER was not a concern for any surface, owing to the advantageous adsorption of CO on each TMP. VP is designated the most active surface for CO reduction at a minimum potential of −0.69 V. VP facilitates the conversion of formaldehyde, methanol, and methane at this adjustable potential. This work presents distinct chemical pathways and a method to mitigate carbon footprints.

## Figures and Tables

**Table 1:** Summary of CO adsorption free energies at different available sites.

TMPs	CO Adsorption Free Energy at Different Available Sites of TMPs	
	M_TOP_	P_TOP_
**CrP**	Unstable	Unstable
**HfP**	−0.2779	1.8163
**NbP**	0.4604	1.1827
**TaP**	−0.3649	Desorbed
**TiP**	−0.1303	1.3616
**VP**	−0.2077	Desorbed
**ZrP**	−0.1122	1.5564

## Data Availability

The original contributions presented in the study are included in the article, further inquiries can be directed to the corresponding authors.
